# A median absolute deviation-neural network (MAD-NN) method for atmospheric temperature data cleaning

**DOI:** 10.1016/j.mex.2021.101533

**Published:** 2021-09-25

**Authors:** Oluwafisayo Owolabi, Daniel Okoh, Babatunde Rabiu, Aderonke Obafaye, Kashim Dauda

**Affiliations:** Centre for Atmospheric Research, National Space Research and Development Agency, Anyigba, Nigeria

**Keywords:** Coarse dataset, Data cleaning, Outliers, Observational data gaps, Climate change, Weather observatories, Atmospheric dataset, Surface air temperature, Neural network, MAD-NN

## Abstract

Some of the biggest challenges in climate change arise from bad dataset. To address this issue, we have developed a novel method for cleaning coarse atmospheric dataset; the median absolute deviation-neural network (MAD-NN) method. By combining the median absolute deviation (MAD) technique with neural network training, this method uses a sequence of steps to clean coarse atmospheric dataset and to predict high accuracy dataset for periods when measurements are not available. To demonstrate this method, we used atmospheric temperature data for 17 different observational weather stations across Nigeria. In brief:

• We developed a novel method for generating consistent data stream from coarse dataset.
• The MAD-NN method can be used to fill observational data gaps and remove spikes in data.
• This method is specifically useful for weather observatories with coarse atmospheric data, as well as increasing the credibility of scientific findings.

## Graphical abstract

**Figure:**
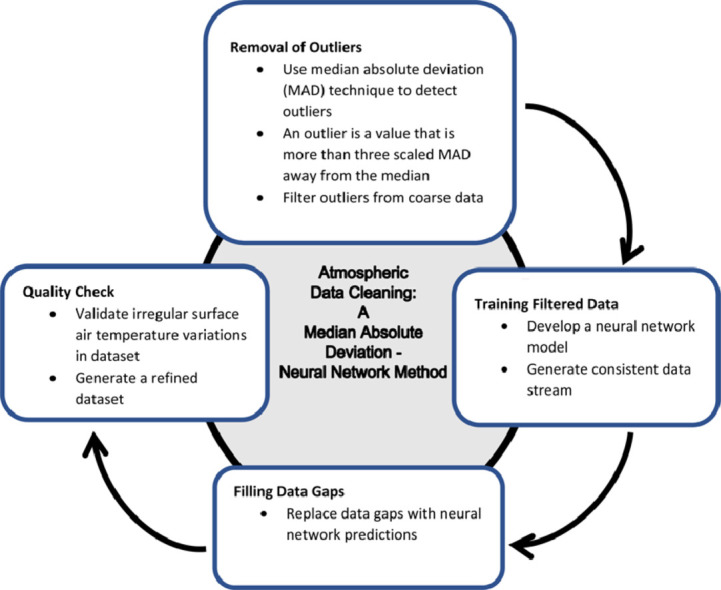

Specifications Table

**Table:** 

Subject Area:	Environmental Science
More specific subject area:	Atmospheric Physics
Method name:	A Median Absolute Deviation-Neural Network (MAD-NN) Method for Atmospheric Temperature Data Cleaning
Name and reference of original method:	None
Resource availability:	The dataset is available on request at https://carnasrda.com/trodan1/

## Background

Concerted efforts on climate change are often hinged on the weather of the Earth's lower atmosphere and their adverse effects in terms of heavy rainfall that causes flood and mudslides, droughts threatening to drive some farmers out of business and the cascading effects of wildfires in many regions of the world. However, some of the biggest challenges in climate change are related to the issues of bad dataset from weather observatories. In fact, quantifying the economic impacts of climate change and the corresponding public health costs and wastages require combing large volumes of datasets in thousands of gigabytes. This explains why using clean dataset to broadcast timely numerical weather forecasts to the public, climate monitoring and environmental assessment of the impacts of weather events are critical in minimizing such wastages and impacts.

Unfortunately, the real-time atmospheric dataset needed to carry-out these socially beneficial weather forecasts and environmental assessment are many times hampered by bad data resulting from incessant power failure, instrument malfunctioning/damage and spikes in data, including data gaps, inaccurate data measurements and associated costs of maintenance of weather observatories. Plus, the allocation of funds for installation and maintenance of weather observatories in the present prevailing socio-economic situation of many developing countries is a recurring challenge. Moreover, manipulating bad dataset can be incredibly challenging with various degrees of difficulties in working with such data [1,2].

To overcome these difficulties, this project is providing a median absolute deviation-neural network (MAD-NN) method for cleaning atmospheric datasets. Cleaning of these datasets involves identifying incomplete, incorrect, inaccurate, or irrelevant parts of the data and then replacing the dirty or coarse data with more appropriate ones. The new dataset generated from this data cleaning method will provide refined atmospheric dataset that will heighten the fidelity of weather forecasts and will therefore help with predicting climate and extreme weather events, and also improve our understanding of atmospheric dynamics. The MAD-NN technique was illustrated in this work using atmospheric surface air temperature data. We note however that the same technique can be applied to other atmospheric parameters like atmospheric pressure, humidity, solar radiation etc.

## Method

The data we adopted to test the MAD-NN technique, and the sequence of steps involved in the technique are explained in the following paragraphs.

**Fig. 1:**
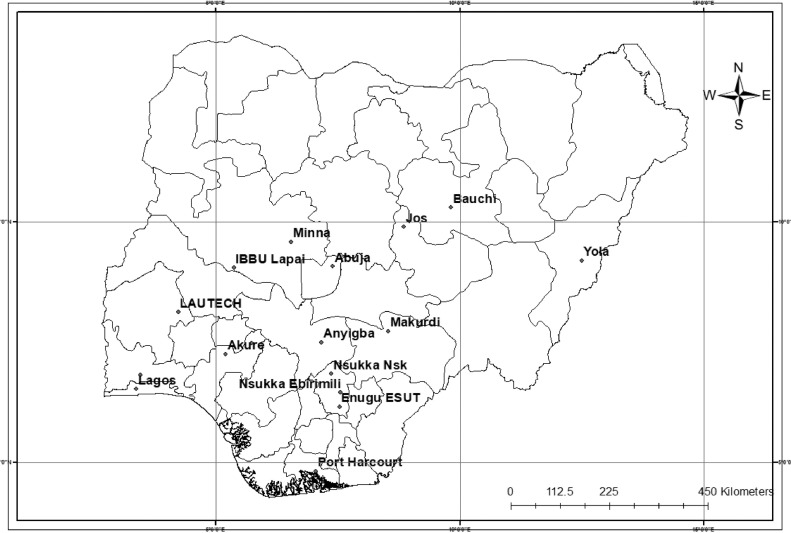
Location of TRODAN weather observatories.

The technique has been applied for cleaning surface air temperature data obtained from the Tropospheric Data Acquisition Network (TRODAN) measurements. TRODAN is an acronym that is adopted to denote measurements from lower atmospheric equipment at the Centre for Atmospheric Research (CAR), Kogi State University Campus, Anyigba, Nigeria. Dataset from this equipment have a time resolution of 5 min, with time range spanning from 0 to 55 min. For each day of full measurements, we therefore expect to have 24*12*17 (=4896) surface air temperature data points, since the surface air temperature data was retrieved from 17 weather observatories in Nigeria. For a year, the number grows to 1,787,040 data points. As at the time of starting this work, the surface air temperature data spanned the period from year 2007 to 2019. Further information about the TRODAN data and how to access it can be found at https://carnasrda.com/trodan1/ [3]. The observatories and the duration of data available from them are shown below in Table 1. Fig. 1 illustrates the geographical location of the weather observatories.

**Table 1:** TRODAN data observatories and period of data available from them.

Station Name	Start Year	End Year
Abuja	2007	2012
Akungba	2008	2011
Akure	2010	2013
Anyigba	2011	2017
Bauchi	2013	2017
Enugu ESUT	2012	2015
IBBU Lapai	2012	2016
Jos	2008	2017
Lagos	2007	2019
LAUTECH	2012	2016
Makurdi	2008	2017
Minna	2008	2017
Nsukka Ebirimili	2008	2017
Nsukka Nsk	2007	2017
Port Harcourt	2008	2017
Redeemers	2010	2011
Yola	2009	2016

**Step 1:** Removal of outliers-the MAD technique.

**Fig. 2:**
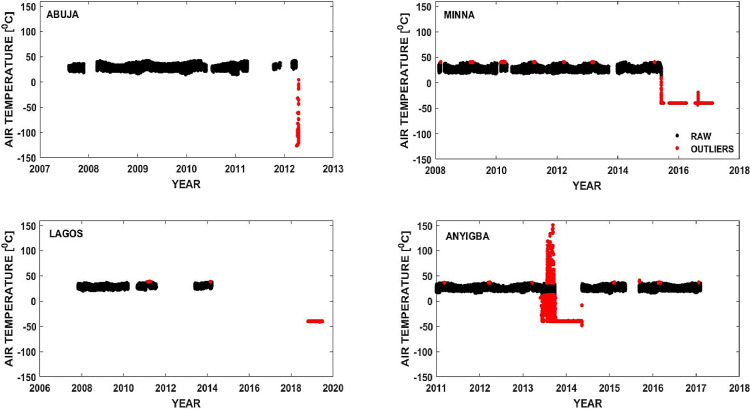
Plots of coarse surface air temperature dataset indicating the outliers in the dataset for Abuja, Minna, Lagos and Anyigba.

For surface air temperature data obtained from a given observatory, an outlier is determined by a value that is more than three scaled median absolute deviations (MAD) away from the median. The value of three is chosen based on the 3-sigma rule which is the best-known criterion to detect an outlier [4]. The threshold value of three is also most commonly used because it has been severally shown to be an effective value for outlier detection [5,6]. Specifically, this value of three is set as the default in MATLAB's implementation of the outlier detection function [7]. To remove the outliers, the MAD technique [8,9] is implemented in a MATLAB script that reads in the raw surface air temperature data in 5 min resolution, and then detects outliers in the dataset by using the MATLAB function named ‘isoutlier’. Fig. 2 illustrates the capacity of the function to correctly detect outliers in the dataset. The points detected as outliers are shown in red color, in Fig. 2. The figure precisely contains surface air temperature dataset from Abuja, Minna, Lagos and Anyigba observatories. In this step of the data cleaning process, the red color data points (representing data detected as outliers) are removed, while the black data points (representing ‘good’ candidates) are included in the preserved dataset. In Fig. 2, there are few points marked in red on the peaks of the concentrated data which are detected as outliers by the algorithm used, whereas they may not actually be outliers. This limitation of the algorithm does not have a significant effect on the results of this work because, at worst case scenario, the data removed represents an insignificant amount of the entire dataset. The 3-Sigma rule typically retains nearly all (∼99.73%) of the entire dataset [4].

**Step 2:** Using neural networks to train preserved data.

After filtering the data to remove the outliers using the MAD technique, the method of artificial neural network is applied to train the preserved data in time series. The feedback forward back-propagation neural network training technique [10,11] was used. In recent times, previous studies by [12], [13], [14] have also used neural networks to train atmospheric dataset collected from the region, and their results indicate that neural networks are good candidates for atmospheric modeling. In particular, [15] posits that the neural network approaches are more accurate than traditional, regression-based approaches. The Levenberg-Marquardt (LM) back-propagation algorithm [16] was used for training. The algorithm is suitable for the training due to its speed and efficiency in learning [13,^17^,18]. References [19], [20], [21] contain more general information on neural networks.

**Fig. 3:**
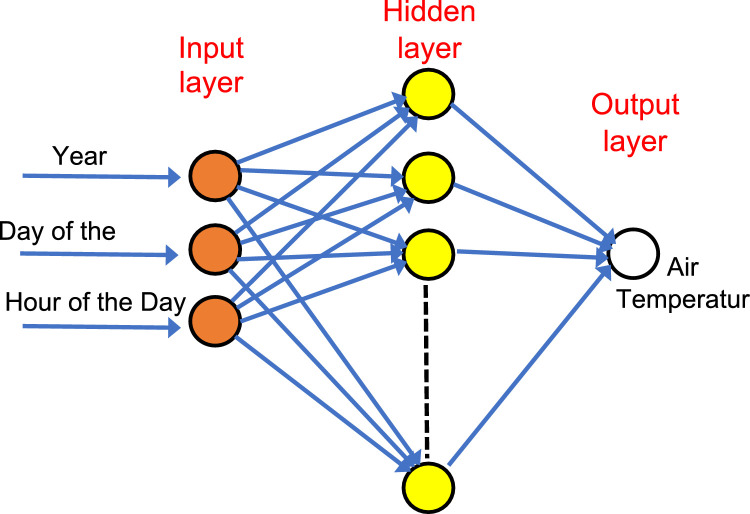
Schematic diagram of neural networks executed in this work.

Fig. 3 is a schematic diagram illustrating the architecture of neural network used in this work. As illustrated in Fig. 3, three input layer neurons were used to enable the networks learn time-series variations of the surface air temperature target. These input neurons are Year, Day of the Year, and Hour of the Day. The ‘Hour of the Day’ input enables the networks to learn diurnal variations of the surface air temperature, the ‘Day of the Year’ input enables the networks to learn seasonal variations associated with the surface air temperatures, and the ‘Year’ input enables the networks to learn the long-term year-to-year variations associated with the surface air temperatures.

Before initializing the neural network training, the entire preserved data was randomly split into three sets: 70% was kept for training, 15% was kept for validation, and the remaining 15% was kept for testing. The MATLAB implementation of the LM back-propagation algorithm, named ‘trainlm’ was used. This algorithm was used to adjust weight matrices and bias vectors in a direction that minimizes the network prediction errors; as training progresses, the weight matrices and bias vectors are adjusted in different training iterations aimed at minimizing the network prediction errors. During each iteration, the validation dataset is used to check when there is no further progress in the generalization. When this happens, the validation check is completed and the training stops.

To decide an appropriate number of neurons for the hidden layer, we trained 20 exactly identical networks that differed only in the number of hidden layer neurons assigned to them, starting from 1 to 20 in steps of 1. Using criteria of minimizing the network prediction errors, we considered the network that gave the least root-mean-square error (RMSE), when simulated for the test dataset, as the most appropriate for consequent surface air temperature predictions.

**Fig. 4:**
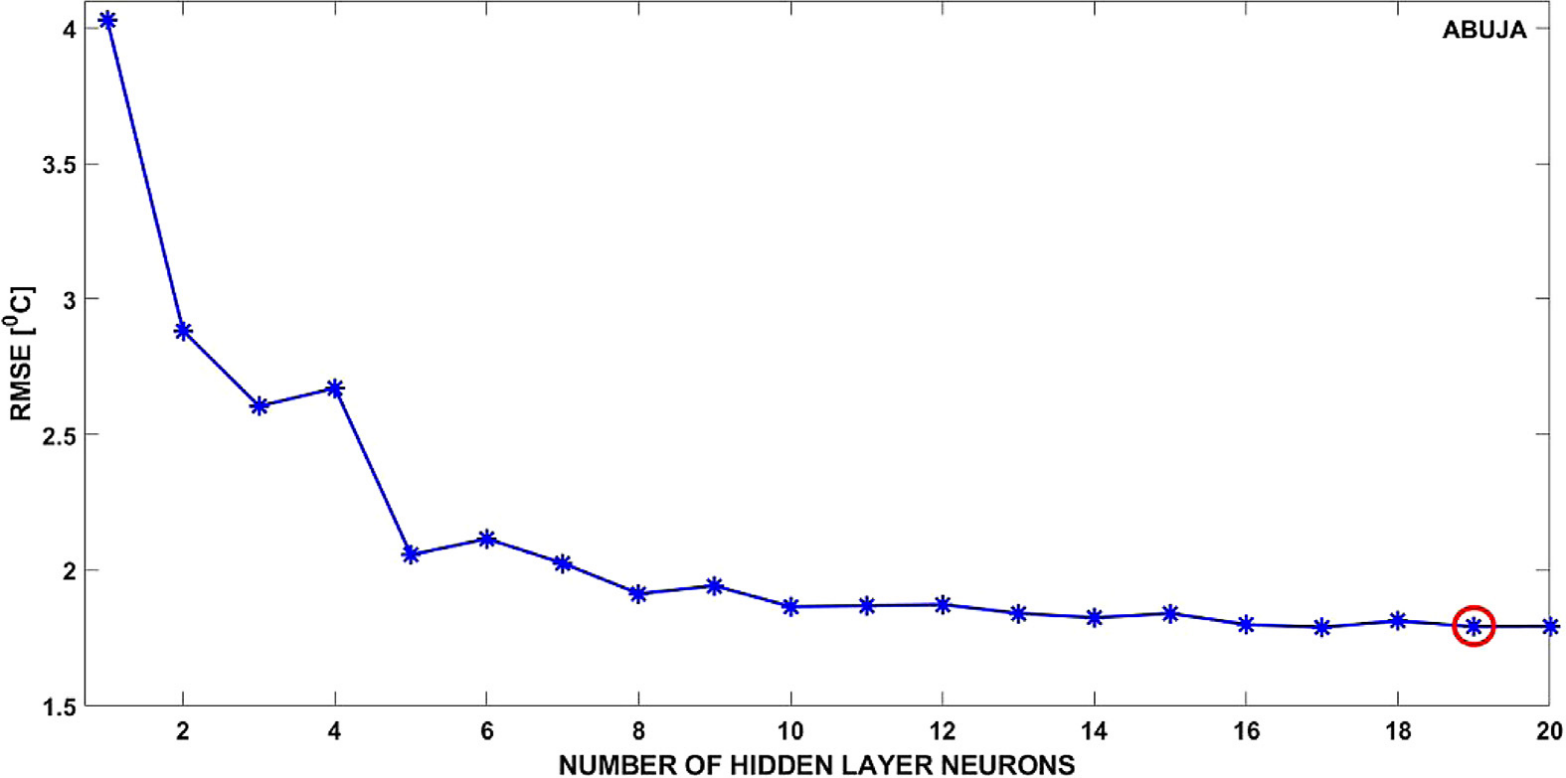
RMSEs for the 20 different neural networks trained in the final neural network for surface air temperature predictions at Abuja.

As further clarification, we have included a plot (Fig. 4) showing variation of the RMSEs with the number of hidden layer neurons for the Abuja observatory. The least RMSE (1.79 °C) was obtained when the number of hidden layer neurons is 19, and this is the network we have used for the Abuja station (number of hidden layer neurons = 19). The following explanation is a justification for limiting the number of hidden layer neurons tested to 20. It is evident (for example, in Fig. 4) that while varying the number of hidden layer neurons from 1 to 10, there is conspicuous change of the RMSE (from 4.03 to 1.87 °C). However, the RMSE no longer changes significantly after the number of neurons exceeds 10. Less than the number where the RMSE no longer changes significantly can lead to under-training, while an excessive number can lead to over-training [22]. Either scenario can lead to bad generalization by the trained network [23]. For these reasons, we avoided using an excessive number of hidden layer neurons. Hence, we benchmark our number of hidden layer neurons to vary from 1 to 20, during which the network with the number of neurons corresponding to the least RMSE is considered as optimal.

**Step 3:** Replacing observational data gaps with neural network predictions and smoothing.

**Fig. 5:**
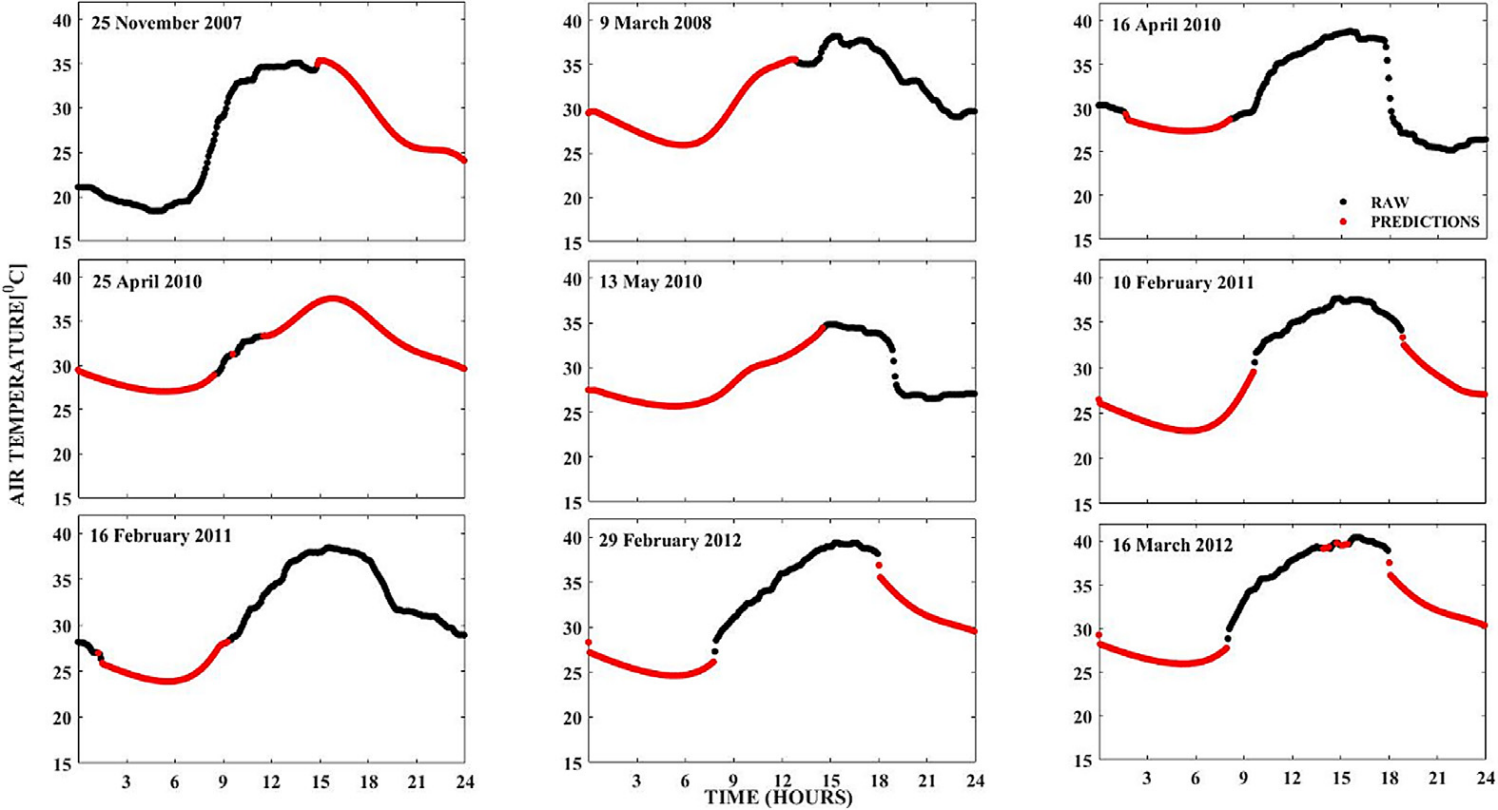
Plots of surface air temperature data gaps being replaced with neural network predictions for the Abuja observatory.

Observational data gaps could be due to gulf in data points created by the removal of outliers or spikes as carried out in **step 1**. They can also be due to power failure or instrument bias as mentioned in the background section. The purpose of this phase of the method is to replace observational gaps resulting from any of these reasons with predictions made by the neural network obtained in **step 2**. This is to ensure that there are data values for all instances of the time resolution associated with the TRODAN measurements. Finally, to smoothen transitions between the TRODAN measurements and the neural network predictions, 2-dimensional median filtering was applied at the interface between the two datasets. Fig. 5 illustrates how the neural network predictions are used to provide complete profiles for some days in which there are data gaps for the Abuja observatory.

The black dots represent ‘actual’ TRODAN measurements of the surface air temperature data, while the red dots represent the neural network predictions. It is evident from the plots in Fig. 5 that the neural network predictions provided fairly accurate representations for the expected TRODAN measurements that are missing. As a measure of the typical errors of the neural network predictions with respect to the actual measurements, we computed the RMSEs between the neural network predictions and the actual measurements for a 15% randomly selected test dataset which was excluded from dataset used for the training. The results showed that the RMSEs for all the stations were consistently less than 2°C, which is in agreement with similar RMSEs obtained in the work of [13]. Specifically, for the Abuja station, which is illustrated in this paper, the RMSE was 1.77°C. This value provides an indication of the confidence level for the neural network predictions. The typical occurrence times of the minimum and maximum surface air temperatures at dawn (between 03:00 and 05:00 LT) and in late afternoon (between 13:00 and 16:00 LT) are also replicated by the neural network predictions [24], confirming the fidelity of this method for surface air temperature measurements.

As a way of showing an example of the number of observational gaps that have been replaced by neural network predictions, we presented in Table 2, a statistical illustration of **step 3** for the Abuja location. The first three columns represent the year, month, and day of the year, respectively. The last column indicates the number of data gaps that have been replaced by the predictions.

**Table 2:** The number of data gaps replaced with neural network predictions for the Abuja observatory, between 2007 and 2010.

Year	Month	Day	Sum (No. of Predictions out of 288)
2007	8	8	154
2007	11	25	110
2008	3	4	274
2008	3	6	245
2008	3	9	155
2008	10	29	1
2010	4	16	77
2010	4	17	107
2010	4	18	100
2010	4	19	88
2010	4	20	89
2010	4	23	21
2010	4	24	97
2010	4	25	252
2010	4	26	266
2010	4	27	260
2010	4	28	283
2010	4	29	272
2010	4	30	280
2010	5	1	271
2010	5	2	283
2010	5	3	284
2010	5	4	266
2010	5	6	285
2010	5	7	285
2010	5	8	285
2010	5	9	270
2010	5	13	173
2010	5	30	68
2010	7	12	206

**Step 4:** Quality check.

**Fig. 6:**
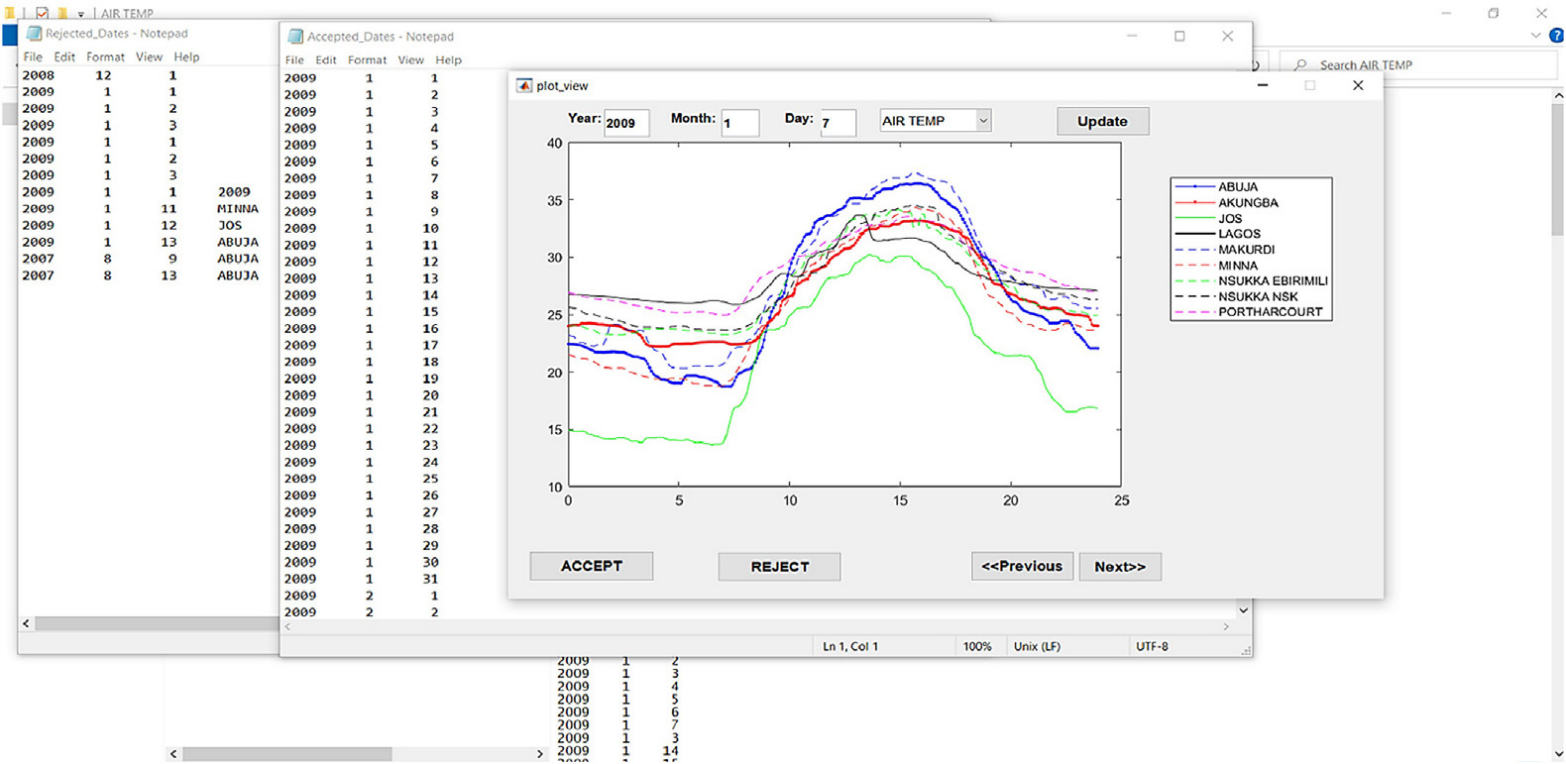
An interface that performs a quality check for the sample air temperature data.

To validate the MAD-NN method, we built an interface as shown in Fig. 6 below, to carry out a final examination of the sample surface air temperature data for the different 17 weather observatories in Nigeria.

**Fig. 7:**
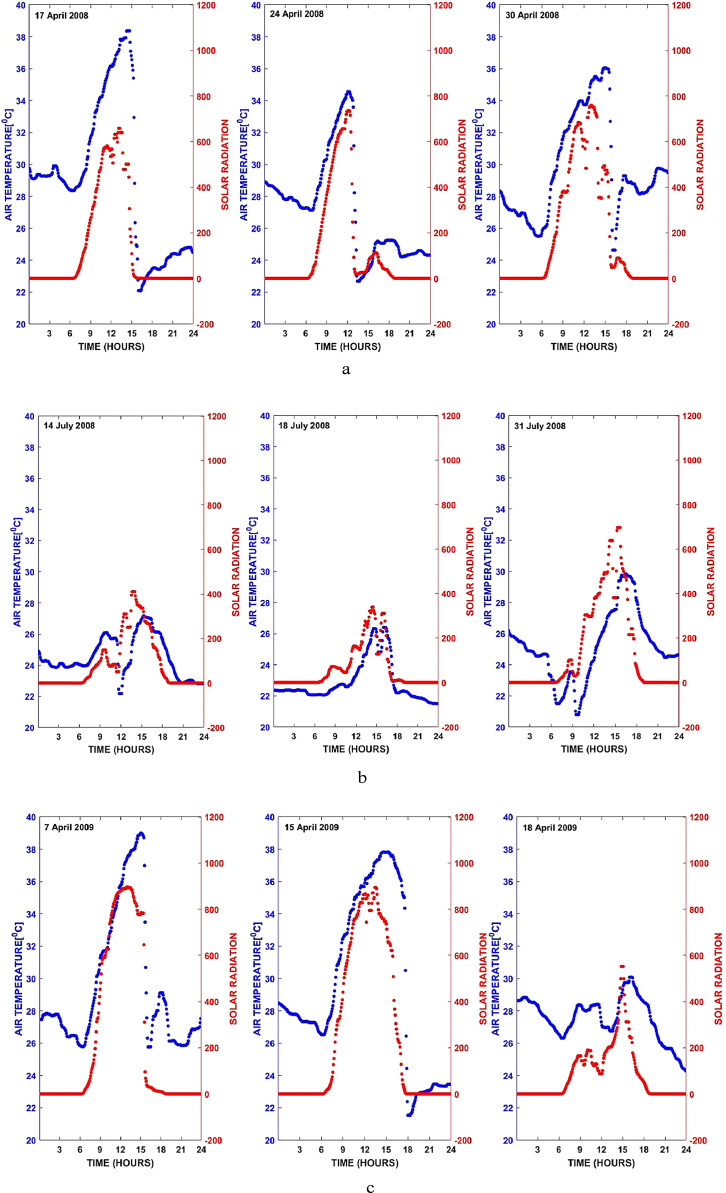
(a) Time series plot of surface air temperature and its corresponding solar radiation measurements. (b) Sample format as Fig. 7a but different dates. (c) Sample format as Fig. 7a but different dates.

Remarkably, during this quality check, we noticed some irregular diurnal surface air temperature variation with steep temperature drops occurring during times like late afternoon hours when temperatures should remain high. To check for the correctness of such data, we develop a hypothesis that the presence of clouds and/or rainfall could significantly block solar radiation from reaching the Earth, and as such could be responsible for such drops in temperature. For data as these, we first verify that co-located solar radiation measurements exhibit similar patterns, and then we accept them. It is important to emphasize at this point that solar radiation measurements are a part of the TRODAN measurements. To apply this hypothesis, we retrieved solar radiation measurements corresponding to the surface air temperature measurements in the foregoing scenario. Fig. 7a–c depicts the example of such irregular surface air temperature variations that we accepted based on the solar radiation hypothesis. While the x-axis represents the time of the day in hours, the right and left hand side of the y-axis denotes the surface air temperatures and solar radiations, respectively.

## Conclusion

We have used a potentially coarse surface air temperature dataset from TRODAN to experiment a unique data cleaning technique, the median absolute deviation-neural network (MAD-NN) method. The dataset span periods from year 2007 to 2019, with over twenty million data points, covering 17 weather observatories in different locations in Nigeria. While developing the method, we have pinpointed steps that can be adopted to clean any ‘dirty’ atmospheric data set: removal of outliers, training neural network to mimic the raw dataset, replacing observational data gaps with neural network predictions and carrying out quality checks to validate the steps involved in the method. By filling observational data gaps and removing the outliers in a dataset, this method helps to increase the data accuracy, availability, and even to predict future dataset. The method promises to accelerate observational and modeling efforts at all stages of atmospheric research, particularly in regions with coarse dataset from weather observatories and where atmospheric research is still nascent. Data generated from this method can be used for environmental weather forecast, climate and meteorological monitoring.

## Declaration of Competing Interest

The authors declare that they have no conflict of interests.
